# *AtERF71*/*HRE2*, an Arabidopsis AP2/ERF Transcription Factor Gene, Contains Both Positive and Negative *Cis*-Regulatory Elements in Its Promoter Region Involved in Hypoxia and Salt Stress Responses

**DOI:** 10.3390/ijms23105310

**Published:** 2022-05-10

**Authors:** Hye-Yeon Seok, Huong Thi Tran, Sun-Young Lee, Yong-Hwan Moon

**Affiliations:** 1 Korea Nanobiotechnology Center, Pusan National University, Busan 46241, Korea; seokhyeon@pusan.ac.kr (H.-Y.S.); symoonlee@pusan.ac.kr (S.-Y.L.); 2 Department of Integrated Biological Science, Pusan National University, Busan 46241, Korea; tranhuongpt90@gmail.com; 3 Department of Molecular Biology, Pusan National University, Busan 46241, Korea; 4 Institute of Systems Biology, Pusan National University, Busan 46241, Korea

**Keywords:** Arabidopsis, *cis*-regulatory element, ERF-VII, HAT22/ABIG1, HD-Zip II, HRE2, hypoxia, salt stress

## Abstract

In the signal transduction network, from the perception of stress signals to stress-responsive gene expression, various transcription factors and *cis*-regulatory elements in stress-responsive promoters coordinate plant adaptation to abiotic stresses. Among the AP2/ERF transcription factor family, group VII ERF (ERF-VII) genes, such as *RAP2.12*, *RAP2.2*, *RAP2.3*, *AtERF73*/*HRE1*, and *AtERF71*/*HRE2*, are known to be involved in the response to hypoxia in Arabidopsis. Notably, *HRE2* has been reported to be involved in responses to hypoxia and osmotic stress. In this study, we dissected *HRE2* promoter to identify hypoxia- and salt stress-responsive region(s). The analysis of the promoter deletion series of *HRE2* using firefly *luciferase* and *GUS* as reporter genes indicated that the −116 to −2 region is responsible for both hypoxia and salt stress responses. Using yeast one-hybrid screening, we isolated HAT22/ABIG1, a member of the HD-Zip II subfamily, which binds to the −116 to −2 region of *HRE2* promoter. Interestingly, HAT22/ABIG1 repressed the transcription of *HRE2* via the EAR motif located in the N-terminal region of HAT22/ABIG1. HAT22/ABIG1 bound to the 5′-AATGATA-3′ sequence, HD-Zip II-binding-like *cis*-regulatory element, in the −116 to −2 region of *HRE2* promoter. Our findings demonstrate that the −116 to −2 region of *HRE2* promoter contains both positive and negative *cis*-regulatory elements, which may regulate the expression of *HRE2* in responses to hypoxia and salt stress and that HAT22/ABIG1 negatively regulates *HRE2* transcription by binding to the HD-Zip II-binding-like element in the promoter region.

## 1. Introduction

Abiotic stresses have been shown to regulate the expression of genes with various functions in a variety of plants [1]. In the signal transduction network, from the perception of stress signals to stress-responsive gene expression, various transcription factors and *cis*-regulatory elements in the stress-responsive promoters are involved in the adaptation of plants to abiotic stresses. Transcription factors can control the expression of many target genes via the specific binding to the *cis*-regulatory element in the promoters of the respective target genes [2]. Several major transcription factor families that are activated in response to abiotic stresses have been identified in Arabidopsis (*Arabidopsis thaliana*), such as AP2/ERF, bZIP, zinc finger, WRKY, MYB, bHLH, and NAC families [3,4].

The AP2/ERF family is a large group of plant-specific transcription factors with 145 members in Arabidopsis, and these 145 genes are classified into the following four subfamilies: AP2, ERF, DREB/CBF, and RAV subfamilies [5]. Members of the ERF and DREB/CBF subfamilies are divided into ten groups from I to X [5]. In particular, the importance of group VII (ERF-VII) genes, such as *RAP2.12*, *RAP2.2*, *RAP2.3*, *AtERF73*/*HRE1*, and *AtERF71*/*HRE2*, in response to hypoxia has been demonstrated [6]. In response to hypoxia, RAP2.12 and RAP2.2 bind to a *cis*-regulatory element, HRPE, and activate downstream genes such as *LBD41* and *PCO1* under hypoxic conditions, whereas HRE1 and HRE2 bind to the GCC box [7,8,9,10].

The expression of ERF-VII genes is regulated at transcriptional and/or post-transcriptional levels. Post-transcriptional regulation of ERF-VII is mediated by N-degron pathway-targeted sequences in their N-terminal regions [6]. In terms of transcriptional regulation, it has recently been reported that *RAP2.2* is transactivated by WRKY33 and WRKY12 under hypoxic conditions via the W-box, 5′-AGTCAA-3′, present in its promoter, while WRKY33 and WRKY12 do not regulate the other four ERF-VII genes [11]. In transcriptional regulation of ERF-VII genes, upstream regulatory transcription factor(s) and responsive *cis*-regulatory element(s) in the promoters of ERF-VII genes, except *RAP2.2*, have not yet been studied.

Homeodomain-leucine zipper (HD-Zip) family is unique to plants, and is characterized by the presence of a homeodomain closely linked to a leucine zipper motif [12]. A total of 48 HD-Zip genes have been identified in Arabidopsis and grouped into four subfamilies: HD-Zip I to HD-Zip IV. Each of the four subfamilies can be distinguished by elevated conservation within the HD-Zip domain, the presence of additional conserved motifs, and specific intron and exon positions [12,13]. HD-Zip proteins are known to control key developmental and environmental responses. *AtHB7* and *AtHB12* function as negative regulators of abscisic acid (ABA) response in Arabidopsis [14]. *AtHB13* is positively regulated by low temperature, drought, and salinity and overexpression of *AtHB13* confers cold, drought, and broad-spectrum disease resistance [15,16,17]. *AtHB2*, *HAT1*, *HAT2*, *HAT3*, and *AtHB4* are rapidly induced by changes in the red/far-red ratio light and promote shade avoidance, a process regulated at multiple levels by auxin [18,19]. In addition, these five genes also play crucial roles in several auxin-regulated developmental processes, including apical embryo patterning, lateral organ polarity, and gynoecium development, in a white light environment [13,20,21,22,23,24].

HD-Zip proteins are transcription factors that function as positive or negative regulators of gene expression [12]. Among these, HD-Zip II subfamily consists of 10 members, and most HD-Zip II proteins contain the LxLxL type of ERF-associated amphiphilic repression (EAR) motif at their N-terminus [13]. Indeed, some HD-Zip II proteins, such as AtHB2, HAT1, HAT2, and AtHB4, function as negative regulators [25,26,27,28]. In contrast, HD-Zip III proteins, such as REV, PHB, and PHA, act as positive regulators of gene expression [29,30,31]. Interestingly, REV transactivates HD-Zip II genes such as *AtHB2*, *HAT2*, *HAT3*, and *AtHB4*, and is involved in the shade avoidance response [23,29]. Recently, it has been reported that *HAT22*/*ABIG1*, a member of the HD-Zip II subfamily, is required for ABA-mediated growth inhibition under drought conditions [32]. However, although HAT22/ABIG1 contains the EAR motif at its N-terminus [33], its function as a transcriptional repressor has not yet been studied.

Previously, *HRE2* has been reported to be involved in both the hypoxia and osmotic stress responses [34,35]. *HRE2* transcription increases under hypoxic, salt, and drought stress conditions, and *HRE2*-overexpressing transgenic plants (OXs) are more tolerant to flooding, salt, and drought stresses. In addition, the promoter activity of *HRE2* is increased by hypoxia and salt stress [35]. In this study, we analyzed the promoter of *HRE2* to identify the abiotic stress-responsive region(s). Promoter analysis using two reporter genes has indicated that −116 to −2 region of *HRE2* promoter is responsible for hypoxia and osmotic stress responses and contains both positive and negative *cis*-regulatory elements. In addition, HAT22/ABIG1, a member of HD-Zip II, binds to the −116 to −2 region via a 7-bp negative *cis*-regulatory element to repress the transcription of *HRE2*.

## 2. Results

### 2.1. Hypoxia-Responsive Positive Cis-Regulatory Element(s) of HRE2 Is Located in the −116 to −2 Region of Its Promoter

**Figure 1:**
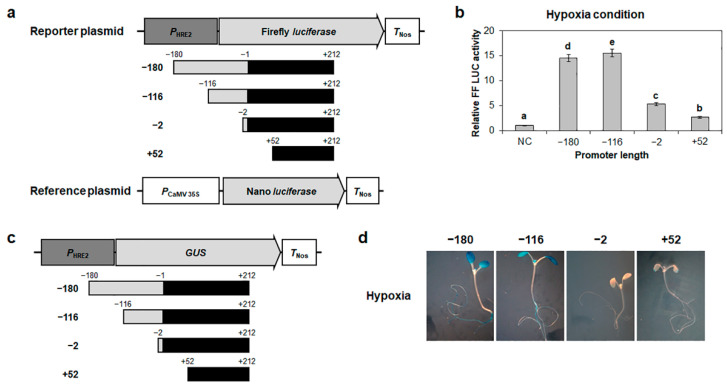
*HRE2* promoter deletion analysis under hypoxia. (**a**) Schematic maps of reporter and reference plasmids for *HRE2* promoter deletion series analysis. (**b**) Relative firefly luciferase activity in Arabidopsis protoplasts. Transformation efficiency was normalized using Nano luciferase activity. Normalized firefly luciferase activity of negative control was set as 1. Empty reporter plasmid was used for the negative control. Data are shown as means ± S.D. (*n* = 3). Different letters display significant differences (*p* < 0.05). NC, negative control. (**c**) A schematic map of vector for *HRE2* promoter deletion series analysis. (**d**) Histochemical GUS assay of Arabidopsis T_2_ transgenic plants carrying the deletion series of *HRE2* promoter at 12 days after germination (DAG) under short-day (SD) conditions. GUS activity was observed in at least 15 transgenic plants for each construct; representative staining results are shown here. In (**a**,**c**), *P*_HRE2_ indicates promoter of *HRE2*.

Previously, we have shown that the 180 bp promoter of *HRE2* responds to hypoxia and salt treatment in Arabidopsis transgenic plants [35]. In this study, we performed a promoter-deletion analysis experiment to identify the hypoxia- and salt-responsive promoter region of *HRE2*. To this end, we generated constructs of firefly *luciferase* genes controlled by the −180 to +212, −116 to +212, −2 to +212, and +52 to +212 regions from the transcriptional start site of *HRE2* promoter (Figure 1a). We then transformed each construct into Arabidopsis protoplasts, which were kept under hypoxic conditions during isolation and then transformation, and measured the firefly luciferase activity driven by the deletion series of *HRE2* promoters. As a result, the −180 and −116 promoters showed high firefly luciferase activity, while −2 promoter showed approximately one-third the activity of that shown by the −180 and −116 promoters (Figure 1b). In addition, +52 promoter showed basal level of firefly luciferase activity (Figure 1b).

We further confirmed this result by measuring GUS activity controlled by the same promoter deletion series of *HRE2* as that used in the firefly luciferase reporter assay (Figure 1c). For this, 12-day-old transgenic plants were subjected to hypoxia, and histochemical GUS assay was performed. As a result, the −180 and −116 promoters showed high GUS activity in the cotyledons, whereas −2 and +52 promoter regions showed no GUS activity under hypoxic conditions (Figure 1d). These results demonstrated that the 115 bp of *HRE2* promoter, namely the −116 to −2 region, includes positive *cis*-regulatory element(s) involved in the response to hypoxia.

**Figure 2:**
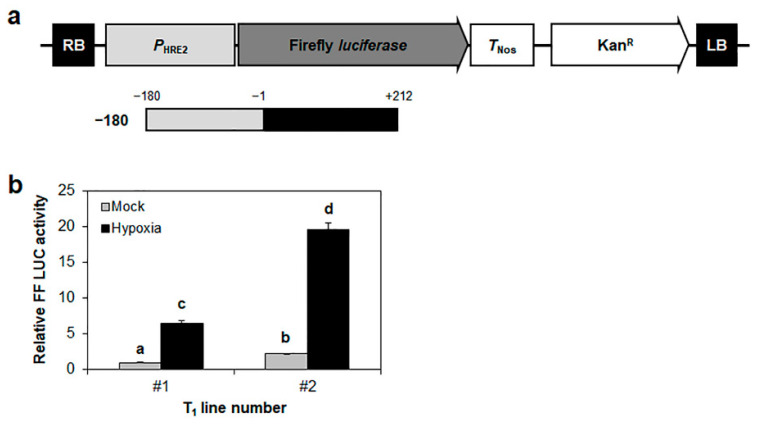
Validation of luciferase assay under hypoxia using Arabidopsis transgenic plants. (**a**) A schematic map of vector for *HRE2* promoter activity analysis. (**b**) Relative firefly luciferase activity in Arabidopsis transgenic plants after being subjected to hypoxia. Hypoxia was induced using N_2_ gas for 12 h. Firefly luciferase activity of mock in line number 1 was set as 1. Data are shown as means ± S.D. (*n* = 3). Different letters display significant differences (*p* < 0.05). Mock indicates normal condition.

Next, we validated the hypoxic response of *HRE2* promoter in Arabidopsis plants. To this end, we generated Arabidopsis transgenic plants harboring firefly *luciferase* gene controlled by the −180 to +212 region from the transcriptional start site of *HRE2* promoter (Figure 2a). We then analyzed firefly luciferase activity in 15-day-old seedlings after hypoxia treatment. We observed that the promoter activity of the −180 promoter was highly increased after hypoxia treatment (Figure 2b), indicating that the −180 promoter of *HRE2* with 5′-UTR is responsive to hypoxia in both protoplasts and plants.

### 2.2. The −116 to −2 Region of HRE2 Promoter Includes Positive Cis-Regulatory Element(s) Responsible for Responses to Salt Stress as Well as Hypoxia

**Figure 3:**
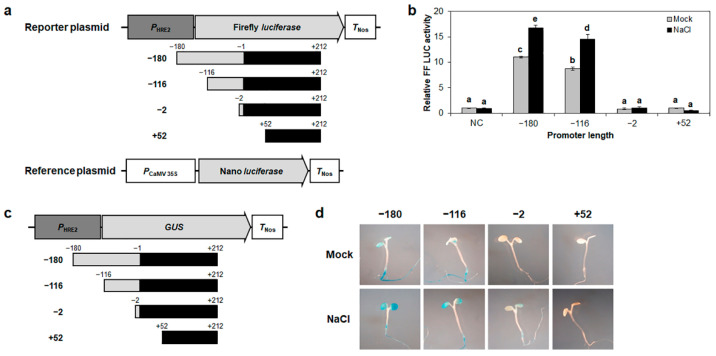
*HRE2* promoter deletion analysis under salt stress condition. (**a**) Schematic maps of reporter and reference plasmids for *HRE2* promoter deletion series analysis. (**b**) Relative firefly luciferase activity in Arabidopsis protoplasts. Transformation efficiency was normalized using Nano luciferase activity. Normalized firefly luciferase activity of negative control in mock was set as 1. Empty reporter plasmid was used for negative control. Data are shown as means ± S.D. (*n* = 3). Different letters display significant differences (*p* < 0.05). (**c**) A schematic map of vector for *HRE2* promoter deletion series analysis. (**d**) Histochemical GUS assay of Arabidopsis T_2_ transgenic plants carrying the deletion series of *HRE2* promoter at 7 DAG under SD conditions. GUS activity was observed in at least 15 transgenic plants for each construct; representative staining results are shown here. In (**b**,**d**), mock indicates normal condition.

*HRE2* is known to respond to salt stress and hypoxia [35]. To identify the salt stress-responsive promoter region of *HRE2*, we transformed the same *HRE2* promoter deletion constructs as those used in the hypoxia-response experiments into Arabidopsis protoplasts under normal or salt stress conditions, and then analyzed the firefly luciferase activity (Figure 3a). The firefly luciferase activities of the −180 and −116 promoters were observed to have increased almost 1.6-fold under salt treatment condition compared to that under normal conditions, while the firefly luciferase activities of the −2 and +52 promoters did not show any response to the salt treatment (Figure 3b).

We also analyzed Arabidopsis transgenic plants harboring *GUS* controlled by the deletion series of *HRE2* promoter (Figure 3c). The results of the histochemical GUS assay showed that the −180 and −116 promoters showed high GUS activity in cotyledons and roots under salt stress conditions, whereas −2 and +52 promoters showed no GUS activity under these conditions (Figure 3d). These results indicated that the −116 to −2 region of *HRE2* promoter is positively involved in the response to salt stress as well as hypoxia.

### 2.3. Reconfirmation of the Positive Response of the −116 to −2 Region of HRE2 Promoter to Hypoxia

**Figure 4:**
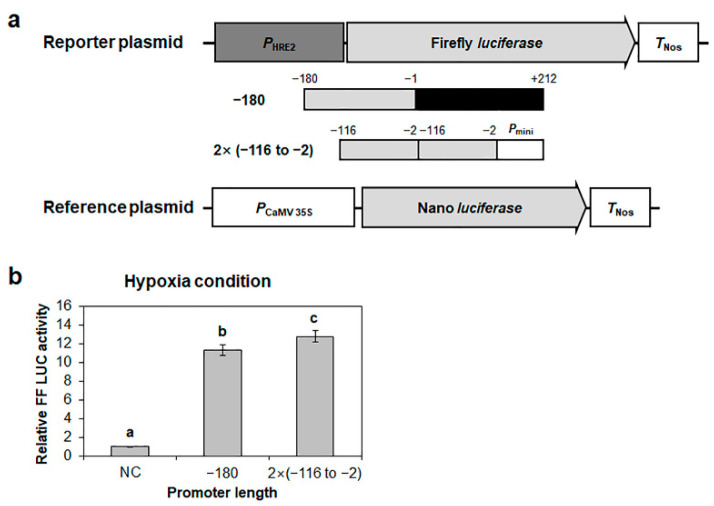
Confirmation of hypoxia-responsive promoter region in *HRE2* promoter. (**a**) Schematic maps of reporter and reference plasmids for *HRE2* promoter activity analysis. (**b**) Relative firefly luciferase activity in Arabidopsis protoplasts. Transformation efficiency was normalized using Nano luciferase activity. Normalized firefly luciferase activity of the negative control was set as 1. Empty reporter plasmid was used for the negative control. Data are shown as means ± S.D. (*n* = 3). Different letters display significant differences (*p* < 0.05).

To reconfirm the positive response of the −116 to −2 region of *HRE2* promoter to hypoxia, we generated a construct containing the firefly *luciferase* gene controlled by tandem repeats of the −116 to −2 region of *HRE2* promoter and transformed it into Arabidopsis protoplasts (Figure 4a). The longest *HRE2* promoter, namely the −180 promoter, was used as the positive control (Figure 4a). Tandem repeats of the −116 to −2 region of *HRE2* promoter showed firefly luciferase activity similar to that of the −180 promoter (Figure 4b), demonstrating that the −116 to −2 region of *HRE2* promoter includes positive *cis*-regulatory element(s) responsible for hypoxia response.

### 2.4. Isolation of Transcription Factor(s) That Bind to the −116 to −2 Region of HRE2 Promoter Using Yeast One-Hybrid Screening

To isolate the transcription factor(s) that bind to the −116 to −2 region of *HRE2* promoter, we performed yeast one-hybrid screening using a cDNA library of Arabidopsis seedlings subjected to hypoxia, in which cDNAs were fused to the GAL4 activation domain (AD). As a result of the screening, a total of 25 positive colonies were obtained from 8.8 × 10^5^ yeast transformants by growth assay using *HIS3* and *ADE2* as reporter genes (Table S1). Plasmid DNAs with AD were isolated from the yeast colonies; we confirmed that the 25 positive plasmid DNAs represented 13 individual genes (Table S2). Interestingly, domain analysis showed that 9 of the 13 genes were homeodomain superfamily genes. Six of these nine genes belonged to the HD-Zip family, while the remaining three belonged to the zinc finger homeodomain (ZF-HD) family (Table S2).

**Figure 5:**
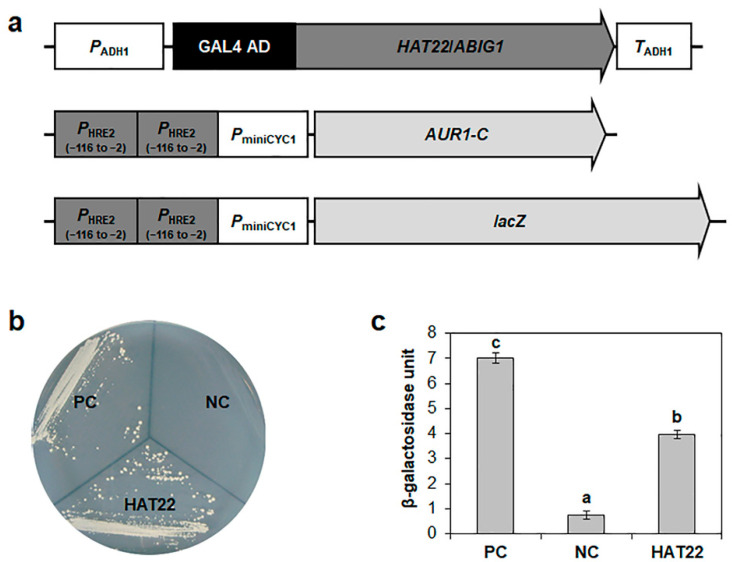
Confirmation of binding of HAT22/ABIG1 to *HRE2* promoter. (**a**) Schematic maps of vectors for yeast one-hybrid assay. *AUR1-C* and *lacZ* reporter genes were used for yeast one-hybrid assay. (**b**) Yeast growth assay. Yeast transformants were grown on SM-Leu/-Ura containing 150 ng/mL Aureobasidin A (AbA). (**c**) Quantitative β-galactosidase orthonitrophenyl-β-D-galactopyronoside (ONPG) assay. β-Galactosidase activities were used for binding activity quantification. The data are shown as means ± S.D. (*n* = 3). Different letters display significant differences (*p* < 0.05). In (**b**,**c**), GCC box and empty vectors were used for positive and negative controls, respectively. PC, positive control; NC, negative control.

We generated constructs including full-length ORFs of the nine homeodomain superfamily genes fused to GAL4 AD, which were then co-transformed into yeasts, together with *AUR1-C* or *lacZ* reporter genes controlled by the tandem repeats of the −116 to −2 region of *HRE2* promoter. Based on the yeast growth and β-galactosidase orthonitrophenyl-β-D-galactopyranoside (ONPG) assays, At4g37790 transactivated the reporter genes most strongly (Figure 5). At4g37790 encodes HAT22/ABIG1, which belongs to class II HD-Zip (HD-Zip II) subfamily. We selected HAT22/ABIG1 for further studies.

### 2.5. HAT22/ABIG1 Is Subcellularly Localized in the Nucleus

**Figure 6:**
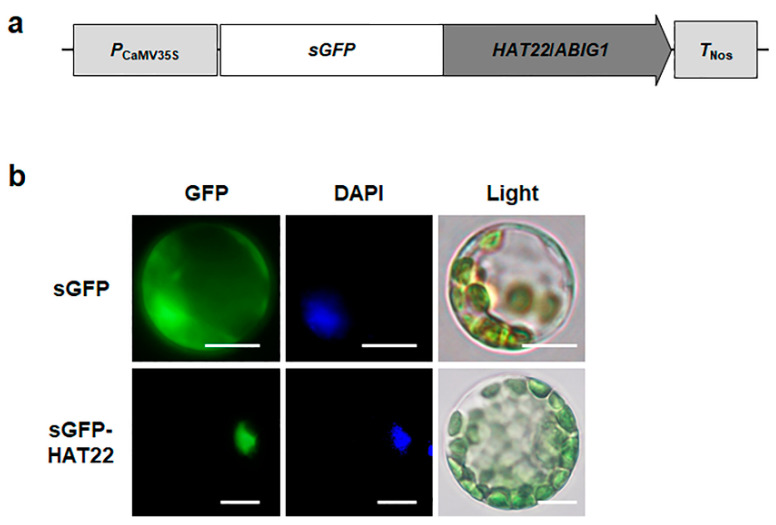
Subcellular localization of HAT22/ABIG1. (**a**) A schematic map of sGFP-fused *HAT22*/*ABIG1* vector. (**b**) Transient expression of sGFP-HAT22/ABIG1 fusion protein in Arabidopsis protoplasts. Left, GFP signal; middle, 4′,6-diamidino-2-phenylindole (DAPI) staining; right, light microscopy images. Scale bars indicate 10 μm.

We investigated the subcellular localization of HAT22/ABIG1 in Arabidopsis protoplasts using an sGFP-HAT22/ABIG1 fusion construct. The GFP signal of the sGFP-HAT22/ABIG1 construct was observed in the nucleus where it overlapped with the 4′,6-diamidino-2-phenylindole signal (Figure 6), indicating that HAT22/ABIG1 functions in the nucleus.

### 2.6. HAT22/ABIG1 Represses HRE2 Transcription via the −116 to −2 Region of HRE2 Promoter

**Figure 7:**
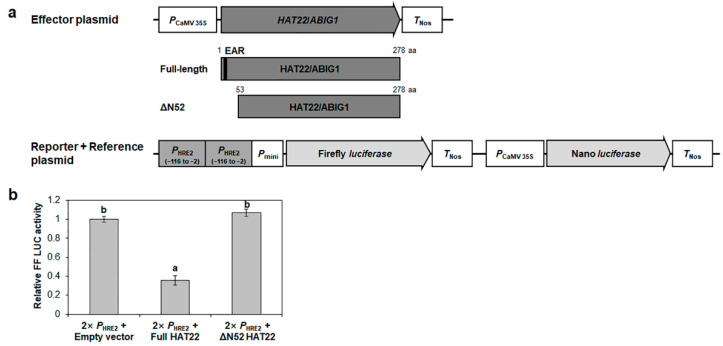
Transrepression assay of HAT22/ABIG1 via *HRE2* promoter. (**a**) Schematic maps of effector and reporter+reference plasmids for the transrepression assay. Black bar in HAT22/ABIG1 indicates EAR motif. (**b**) The relative firefly luciferase activity in Arabidopsis protoplasts. The transformation efficiency was normalized using Nano luciferase activity. The normalized firefly luciferase activity of the negative control was set as 1. The empty effector plasmid was used for the negative control. Data are shown as means ± S.D. (*n* = 5). Different letters display significant differences (*p* < 0.05).

It has been reported that HD-Zip II proteins, such as AtHB2, HAT1, HAT2, and AtHB4, function as transcriptional repressors by means of the EAR motif located in their N-terminal regions [25,26,27,28]. HAT22/ABIG1 also contains an EAR motif at its N-terminus [33], indicating that HAT22/ABIG1 might function as a transcriptional repressor in the regulation of downstream genes. To check the transcriptional repression of *HRE2* by HAT22/ABIG1, firefly *luciferase* gene controlled by the tandem repeats of the −116 to −2 region of *HRE2* promoter was co-transformed with the *HAT22*/*ABIG1* OX construct into Arabidopsis protoplasts (Figure 7a). The firefly luciferase activity with HAT22/ABIG1 was almost one-third of that without HAT22/ABIG1 (Figure 7b), demonstrating that HAT22/ABIG1 represses *HRE2* transcription via the −116 to −2 region of *HRE2* promoter in Arabidopsis plants.

We also tested whether the EAR motif in the N-terminal region of HAT22/ABIG1 is important for its transcriptional repression activity. We generated the *HAT22*/*ABIG1* OX construct (ΔN52 *HAT22*/*ABIG1*), in which 52 aa of the N-terminus of HAT22/ABIG1, including the EAR motif, were deleted. We then analyzed the effect of ΔN52 HAT22/ABIG1 on the firefly luciferase activity controlled by the tandem repeats of the −116 to −2 region of *HRE2* promoter (Figure 7a). The firefly luciferase activity with ΔN52 HAT22/ABIG1 was recovered to the level observed for that without HAT22/ABIG1 (Figure 7b). It was previously reported that HD-Zip II proteins bind to the promoters of downstream genes through homeodomain [13]. Predicted nuclear localization sequences (NLS) of AtHB4, a HD-Zip II protein, is in the homeodomain and the EAR motif-deleted AtHB4 is subcellularly localized in the nucleus [28]. We found that predicted NLS of HAT22/ABIG1 is also in the homeodomain (125–179 aa region) using NLS Mapper (https://nls-mapper.iab.keio.ac.jp/cgi-bin/NLS_Mapper_form.cgi, accessed on 29 April 2022) (data not shown), suggesting that ΔN52 HAT22/ABIG1 is translocated to the nucleus and binds to the −116 to −2 region of *HRE2* promoter. Indeed, GFP signal of the sGFP-ΔN52 HAT22/ABIG1 construct was observed in the nucleus (Figure S2), demonstrating that ΔN52 HAT22/ABIG1 is translocated to the nucleus. Our results together with the predictions indicate that the EAR motif in the N-terminus of HAT22/ABIG1 is important for its repression of *HRE2* transcription.

We further tested the transactivation activity of HAT22/ABIG1 to check whether HAT22/ABIG1 acts as a transcriptional activator. HAT22/ABIG1 was fused to the GAL4 DNA-binding domain (BD) and transformed into yeast. As expected, HAT22/ABIG1 did not show transactivation activity in the yeast growth and β-galactosidase ONPG assays (Figure S3).

### 2.7. HAT22/ABIG1 Represses HRE2 Transcription via 7 bp Conserved Negative Cis-Regulatory Element, 5′-AATGATA-3′, in the −116 to −2 Region of HRE2 Promoter

**Figure 8:**
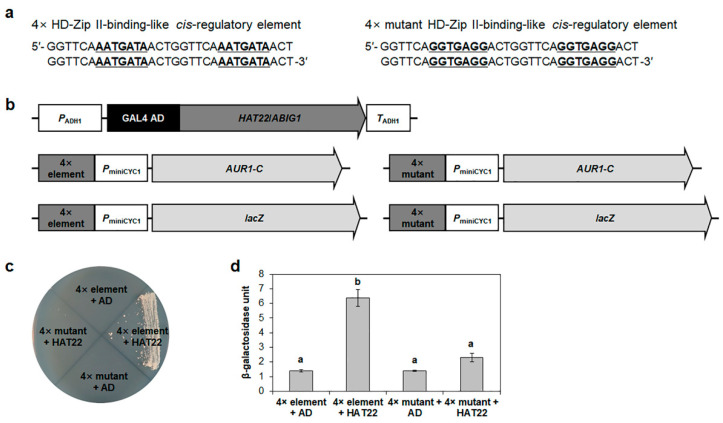
Confirmation of binding of HAT22/ABIG1 to HD-Zip II-binding-like *cis*-regulatory element in yeast. (**a**) Sequences of tandem repeats of HD-Zip II-binding-like *cis*-regulatory element or mutant HD-Zip II-binding-like *cis*-regulatory element for yeast one-hybrid assay. Consensus sequences are underlined. (**b**) Schematic maps of vectors for yeast one-hybrid assay. *AUR1-C* and *lacZ* reporter genes were used for yeast one-hybrid assay. (**c**) Yeast growth assay. Yeast transformants were grown on SM-Leu/-Ura containing 150 ng/mL AbA. (**d**) Quantitative β-galactosidase ONPG assay. β-Galactosidase activities were used for binding activity quantification. Data are shown as means ± S.D. (*n* = 3). Different letters display significant differences (*p* < 0.05). In (**c**,**d**), GCC box and empty vectors were used for positive and negative controls, respectively.

HAT22/ABIG1 is a member of the HD-Zip II subfamily. The HD-Zip II subfamily is known to repress downstream genes via the conserved regulatory sequence, 5′-AAT(G/C)ATT-3′ [13]. Our results showed that *HRE2* promoter contains 5′-AATGATA-3′ (−69 to −63 region) in its hypoxia- and salt-responsive −116 to −2 region (Figure S4). This observation led us to hypothesize that the transcriptional repression of *HRE2* by HAT22/ABIG1 is regulated by the 7 bp sequence. We generated four constructs of *AUR1-C* and *lacZ* reporter genes under the control of four tandem repeats of the 16 bp *HRE2* promoter region, including 5′-AATGATA-3′ or its mutated sequence, 5′-GGTGAGG-3′ (Figure 8a). The constructs were then co-transformed with GAL4 AD-fused HAT22/ABIG1 into yeast (Figure 8b). In the yeast growth and β-galactosidase ONPG assays, 5′-AATGATA-3′ resulted in transactivation by HAT22/ABIG1 in yeast, whereas 5′-GGTGAGG-3′ did not show any transactivation (Figure 8c,d and Figure S5). These results demonstrated that the 7 bp negative *cis*-regulatory element, 5′-AATGATA-3′, plays an important role in the transcriptional regulation by HAT22/ABIG1.

**Figure 9:**
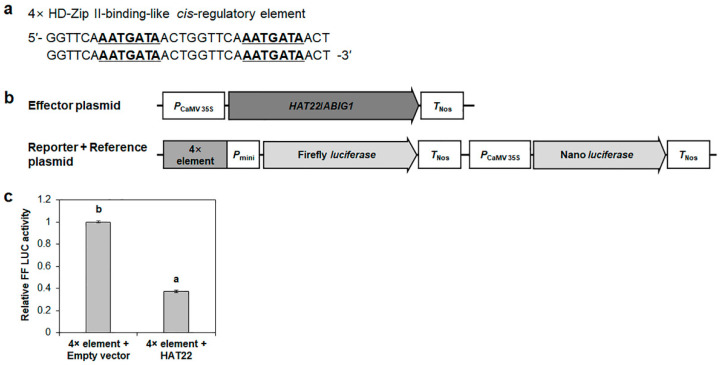
Transrepression assay for HAT22/ABIG1 via HD-Zip II-binding-like *cis*-regulatory element. (**a**) Sequences of tandem repeats of HD-Zip II-binding-like *cis*-regulatory element for transrepression assay. Consensus sequences are underlined. (**b**) Schematic maps of effector and reporter+reference plasmids for the transrepression assay. (**c**) The relative firefly luciferase activity in Arabidopsis protoplasts. The transformation efficiency was normalized using Nano luciferase activity. The normalized firefly luciferase activity of negative control was set as 1. The empty effector plasmid was used for the negative control. Data are shown as means ± S.D. (*n* = 5). Different letters display significant differences (*p* < 0.05).

We also constructed a firefly *luciferase* gene under the control of four tandem repeats of the 16 bp *HRE2* promoter region and co-transformed it with *HAT22*/*ABIG* OX construct into Arabidopsis protoplasts (Figure 9a,b). The results showed that firefly luciferase activity in the presence of HAT22/ABIG1 was almost one-third of that without HAT22/ABIG1 (Figure 9c). These results conclusively indicated that HAT22/ABIG1 transcriptionally represses *HRE2* via the 7 bp negative *cis*-regulatory element, 5′-AATGATA-3′.

### 2.8. HAT22/ABIG1 Is Responsive to Both Hypoxia and Salt Stresses

**Figure 10:**
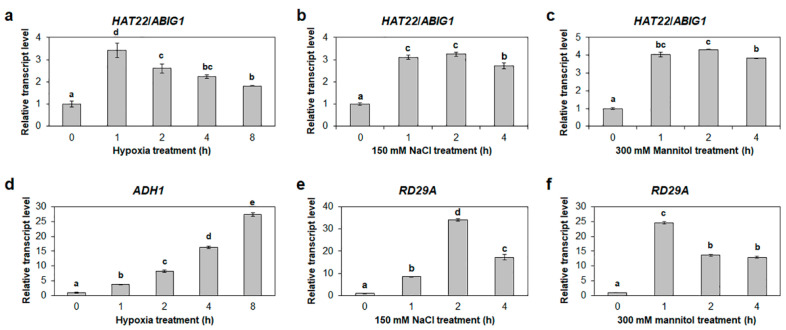
Expression of *HAT22*/*ABIG1* under osmotic stress and hypoxic conditions. (**a**,**b**) Quantitative RT-PCR (RT-qPCR) analysis for *HAT22*/*ABIG1* (**a**) and *ADH1* (**d**) in WT seedling upon treatment with 99.9% N_2_ gas for 0, 1, 2, 4, and 8 h under dark conditions. (**b**,**e**) RT-qPCR analysis for *HAT22*/*ABIG1* (**b**) and *RD29A* (**e**) in WT seedling upon treatment with 150 mM NaCl for 0, 1, 2, and 4 h. (**c**,**f**) RT-qPCR analysis for *HAT22*/*ABIG1* (**c**) and *RD29A* (**f**) in WT seedling upon treatment with 300 mM mannitol for 0, 1, 2, and 4 h. *Glyceraldehyde 3-phosphate dehydrogenase* (*GAPc*) was used for an endogenous reference gene. Transcript levels at 0 h were set to 1. Data are shown as means ± S.D. (*n* = 3). At least three biological replicates showed similar results; representative results are shown here. Different letters display significant differences (*p* < 0.05).

Previously, *HAT22*/*ABIG1* was found to be responsive to drought stress and ABA [32] (Figure 10). However, responses of *HAT22*/*ABIG1* to hypoxia and/or salt stress have not yet been reported. To determine the expression of *HAT22*/*ABIG1* under hypoxic and salt stress conditions, the transcript abundance of *HAT22*/*ABIG1* was examined under these conditions. Quantitative RT-PCR (RT-qPCR) results showed that the expression of *HAT22*/*ABIG1* increased at 1 h after being subjected to hypoxia and then gradually decreased until 8 h after hypoxia treatment (Figure 10). In addition, the expression of *HAT22*/*ABIG1* also increased at 1 h after the treatment with NaCl, and the expression level was maintained up to 4 h after the treatment (Figure 10). Increased expression of *ADH1* and *RD29A*, hypoxia and osmotic-stress marker genes, respectively, confirmed that the hypoxia, NaCl, and mannitol stresses were properly treated (Figure 10). These results indicated that *HAT22*/*ABIG1* is involved in the response to hypoxia and salt stress.

## 3. Discussion

HRE2 is a member of the ERF-VII transcription factor group in Arabidopsis, and the ERF-VII group is well known to be involved in the hypoxia response in plants [6]. The ERF-VII group members in Arabidopsis, namely RAP2.12, RAP2.2, RAP2.3, HRE1, and HRE2, are post-transcriptionally regulated by the N-degron pathway; however, their transcriptional regulation is not well understood [6]. Moreover, signal transduction pathways involving ERF-VII group genes, including upstream transcriptional regulators and downstream genes, have not been well studied. In this study, we identified the *HRE2* promoter region containing hypoxia- and salt stress-responsive positive *cis*-regulatory element(s). In addition, we isolated HAT22/ABIG1 as a transcriptional repressor of *HRE2* transcription in responses to hypoxia and salt stress, and identified a negative *cis*-regulatory element bound by HAT22/ABIG1 in *HRE2* promoter.

We have previously reported that *HRE2* is involved in responses to both hypoxia and salt stress and that the 180 bp promoter of *HRE2* includes positive *cis*-regulatory element(s) responsible for these responses [35]. To elucidate the signal transduction pathway of hypoxia and salt stress responses via *HRE2*, we first analyzed the region of *HRE2* promoter responsible for hypoxia and salt stress responses. The analysis using firefly *luciferase* and *GUS* as reporter genes controlled by deletion series of the 180 bp *HRE2* promoter showed that the −116 to −2 region of *HRE2* promoter includes positive *cis*-regulatory element(s) responsible for both hypoxia and salt stress responses (Figure 1, Figure 2, Figure 3 and Figure 4). We analyzed potential *cis*-regulatory elements in the −116 to −2 region of *HRE2* promoter using PLACE (https://www.dna.affrc.go.jp/PLACE/?action=newplace, accessed on 2 April 2022), software for the analysis of plant *cis*-regulatory element(s) in the promoter. However, we could not find candidate(s) for hypoxia-responsive positive *cis*-regulatory element(s) (data not shown).

Using yeast one-hybrid screening, we isolated HAT22/ABIG1, a member of the HD-Zip II subfamily, which binds to the −116 to −2 region of *HRE2* promoter (Table S2 and Figure 5). It has been well known that HD-Zip II proteins contain LxLxL-type EAR motif in their N-terminus and repress downstream genes by binding to 7 bp conserved regulatory sequences, 5′-AAT(G/C)ATT-3′, in the promoters of the downstream genes [13]. For example, HAT1 directly binds to the target genes of brassinosteroids and functions as a co-repressor together with BES1 [27]. AtHB2 acts as a negative regulator and induces hypocotyl elongation by inhibiting auxin transport inhibitors [36]. Interestingly, the −116 to −2 region of *HRE2* promoter contains 5′-AATGATA-3′ sequence, which is similar to the HD-Zip II-binding 7 bp element (Figure S4). The yeast one-hybrid assay and transrepression assay in Arabidopsis protoplasts showed that HAT22/ABIG1 binds to the 7 bp conserved regulatory sequence and represses the transcription of *HRE2* (Figure 8 and Figure 9). Our results demonstrated that HAT22/ABIG1 represses the transcription of *HRE2* via the 7 bp negative *cis*-regulatory element, 5′-AATGATA-3′, in the −116 to −2 region of *HRE2* promoter in responses to hypoxia and/or salt stress, and that the EAR motif in the N-terminus of HAT22/ABIG1 plays an important role in this transcriptional repression. This is the first report to clarify that the 7 bp negative *cis*-regulatory element is involved in hypoxia and salt stress signal transduction via the HD-Zip II protein HAT22/ABIG1. As the transcriptional regulator(s) that activate *HRE2* transcription remain unidentified in this study, further studies using the −116 to −2 region of *HRE2* promoter are needed to isolate and characterize the transcriptional activators.

Gene expression is tightly regulated by transcriptional activators and repressors. Regulation of the balance between activators and repressors is important for proper gene expression and responses to abiotic stresses [11]. DREB1/CBF proteins transactivate *RD29A* and *COR15A* to lead tolerance to freezing temperature, whereas DEAR1 protein represses *RD29A* and *COR15A* to tightly control during normal growth and development [37]. NAC016 and AtNAP negatively regulate *AREB1* under drought stress, whereas SnRK2.2 positively regulates *AREB1*, resulting in fine-tuning of the spatiotemporal control of drought stress-responsive signaling [38,39]. Our results showed that the −116 to −2 region of *HRE2* promoter contains both positive and negative *cis*-regulatory elements involved in responses to hypoxia and salt stress and that the negative *cis*-regulatory element is bound by HAT22/ABIG1, indicating that the transcription of *HRE2* might be properly regulated by both transcriptional activator(s) and repressor(s).

The ERF-VII group of the AP2/ERF family can be divided into two types, namely, the RAP-type, which includes RAP2.12, RAP2.2, and RAP2.3, and the HRE-type, which includes HRE1 and HRE2 [6]. Recently, it was reported that *RAP2.2* is transactivated by WRKY33 and WRKY12 in the hypoxia response via the W-box, 5′-AGTCAA-3′, in *RAP2.2* promoter. However, *HRE2* and *HRE1* are not regulated by WRKY33 and WRKY12 [11] and our analysis revealed that the *HRE2* promoter does not contain the W-box (data not shown). On the other hand, RAP2.12 and RAP2.2 transactivate downstream genes via HRPE, a hypoxia-responsive *cis*-regulatory element, whereas HRE1 and HRE2 transactivate downstream genes via the GCC box [7,8,9,10]. These results suggest that the RAP-type and HRE-type ERF-VII groups might be involved in separate signal transduction pathways in the hypoxia response.

Taken together, our results demonstrate that the −116 to −2 region of *HRE2* promoter contains both positive and negative *cis*-regulatory elements involved in hypoxia and salt stress responses and that HAT22/ABIG1 transcriptionally represses *HRE2* via 5′-AATGATA-3′ sequence, which is a negative *cis*-regulatory element present in the −116 to −2 region.

## 4. Materials and Methods

### 4.1. Plant Materials and Growth Conditions

All *Arabidopsis thaliana* plants used in this study were of the Columbia (Col-0) ecotype. Arabidopsis seeds preparation, germination, and growth were performed according to previous study [35].

### 4.2. Plasmid Construction

To generate deletion series of *HRE2* promoter, −180 to +212, −116 to +212, −2 to +212, and +52 to +212 regions from the transcriptional start site of *HRE2* were amplified by PCR and cloned into pFGL1495 or pFGL539 fused with firefly *luciferase* or *GUS*, respectively. Two tandem repeats of −116 to −2 region of *HRE2* promoter were cloned into pFGL1437 fused with firefly *luciferase*.

To construct plasmids for the yeast one-hybrid assay, the promoter regions of *HRE2* were amplified by PCR and cloned into pAbAi or pLacZi fused with *AUR1-C* or *lacZ*, respectively. The full-length ORF of HAT22/ABIG1 was amplified by PCR and cloned into pGADT7 in-frame with GAL4 AD.

To generate plasmids for the transrepression assay in Arabidopsis protoplasts, the promoter regions of *HRE2* were amplified by PCR and cloned into pFGL1437 fused with firefly *luciferase*.

The primers for cloning are listed in Table S3.

### 4.3. Generation of Arabidopsis Transgenic Plants

The constructs for expression in Arabidopsis were transformed into *Agrobacterium tumefaciens* strain GV3101 (pMP90) using the freeze–thaw method [40] and then introduced into WT Arabidopsis using the floral-dipping method [41]. Transgenic plants were selected by 50 mg/L of kanamycin in MS plates.

### 4.4. Stress Treatment

For the hypoxia treatment, 10-day-old WT seedlings grown on MS plates were transferred to MS medium-saturated filter paper and then were treated with 99.99% N_2_ gas under dark conditions for 0, 1, 2, 4, and 8 h.

For NaCl and mannitol treatments, 10-day-old WT seedlings grown on MS plates were transferred to filter papers saturated with MS medium containing 150 mM NaCl or 300 mM mannitol and kept for 0, 1, 2, and 4 h.

### 4.5. Histochemical GUS Assay

GUS activity was detected histochemically following a previously described protocol [35].

### 4.6. Protoplast Transformation

Arabidopsis protoplast isolation and transformation were conducted according to Yoo et al. [42].

### 4.7. Dual-Luciferase Assay

Luciferase activity was quantified using the Nano-Glo^®^ Dual-Luciferase^®^ Reporter Assay System (Promega Corp., Madison, WI, USA) and the GloMax^®^-Multi+ Detection System (Promega Corp., Madison, WI, USA), in accordance with the manufacturer’s instructions.

### 4.8. cDNA Library Generation and Yeast One-Hybrid Screening

To generate a hypoxia cDNA library, 7- and 14-day-old seedlings grown under short-day conditions were subjected to hypoxia for 1 and 3 h. Total RNA was isolated using RNAqueous Kit (Invitrogen, Carlsbad, CA, USA) and Plant RNA Isolation Aid (Invitrogen, Carlsbad, CA, USA). Subsequently, cDNA library was generated using Make Your Own “Mate & Plate” Library System (Clontech Laboratories, Inc., Mountain View, CA, USA). The cfu value of the cDNA library was 1.43 × 10^7^. Yeast one-hybrid screening was performed using Matchmaker^®^ Gold Yeast One-Hybrid Library Screening System. pADE2i harboring two tandem repeats of the −116 to −2 region of *HRE2* promoter was used as the bait in yeast one-hybrid screening. cDNA library generation and yeast one-hybrid screening were performed by PanBioNet (http://www.panbionet.com, accessed on 19 February 2019).

### 4.9. Yeast Transformation and Assay

The constructs for the yeast one-hybrid assay were transformed into Y1HGOLD or YM4271. Yeast transformation was performed by the Frozen-EZ Yeast Transformation II^TM^ Kit (Zymo Research Corp., Irvine, CA, USA), in accordance with the manufacturer’s instructions. A quantitative β-galactosidase assay was performed using ONPG as a substrate. The unit of β-galactosidase activity was calculated using the formula 1000 × OD_420_/(OD_600_ × assay time in min × assay volume in mL). Transformants were analyzed using 5-bromo-4-chloro-3-indolyl-β-d-galactopyranoside as a substrate for the β-galactosidase filter assay. The reaction was carried out for 6 h. For the yeast growth assay, transformants were streaked onto synthetic minimal media lacking leucine and uracil containing 150 ng/mL Aureobasidin A (AbA) and incubated for 3–5 days at 30 °C.

### 4.10. RNA Isolation, cDNA Synthesis, and RT-qPCR

Total RNA was isolated by RNAqueous Kit (Invitrogen, Carlsbad, CA, USA) and Plant RNA Isolation Aid (Invitrogen, Carlsbad, CA, USA) in accordance with the manufacturer’s protocol. Two micrograms of total RNA was reverse-transcribed using Moloney murine leukemia virus reverse transcriptase (Promega Corp., Madison, WI, USA). RT-qPCR was performed and analyzed using Power SYBR Green PCR Master mix (Applied Biosystems, Foster, CA, USA), QuantStudio^TM^ 3 real-time PCR system (Applied Biosystems, Foster, CA, USA), and QuantStudio^TM^ Design and Analysis software v.1.4.3 (Applied Biosystems, Foster, CA, USA) in accordance with the manufacturer’s manual. Three independent reactions were conducted for each technical replicate. Two technical replicates were conducted for each biological replicate. The primers for RT-qPCR are listed in Table S4.

### 4.11. Statistical Analysis

Statistical analysis was performed by IBM SPSS Statistics software version 23 (IBM Corp., Armonk, NY, USA) with one-way ANOVA using Tukey’s multiple comparison test.

## Data Availability

The data presented in this study are available in the Supplementary Materials.

## Supplementary Materials

The following supporting information can be downloaded at: https://www.mdpi.com/article/10.3390/ijms23105310/s1.
