# Supplementary material for: AtERF71/HRE2, an Arabidopsis AP2/ERF Transcription Factor Gene, Contains Both Positive and Negative Cis-Regulatory Elements in Its Promoter Region Involved in Hypoxia and Salt Stress Responses
Source: Int J Mol Sci. 2022 May 10;23(10):5310. doi: 10.3390/ijms23105310 (PMC9140466; doi:10.3390/ijms23105310)
Supplement: Supplementary file 1 [file ijms-23-05310-s001.zip › ijms-1696579-supplementary.pdf]

Supplementary data

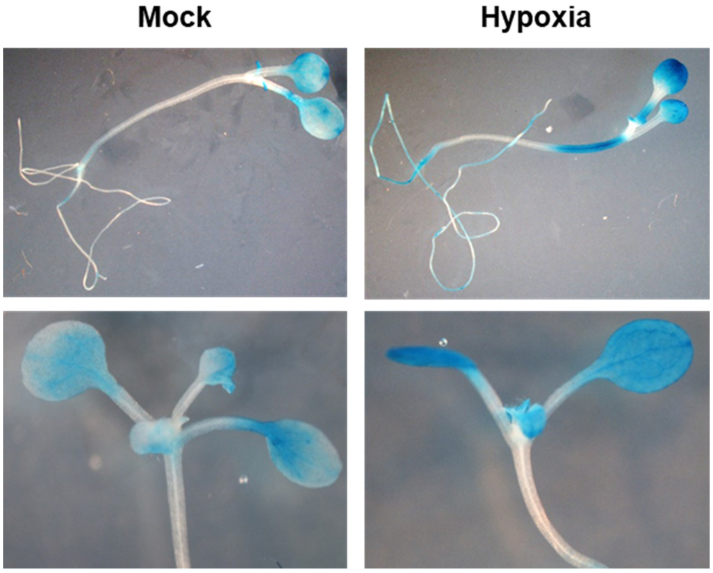

Figure S1. *HRE2* promoter analysis under hypoxic condition. Histochemical GUS assay for Arabidopsis T_2 transgenic plants carrying the -180 of *HRE2* promoter at 12 DAG under hypoxic condition. Hypoxia was induced using 99.9% N_2 gas for 12 h. GUS activity was observed in at least 15 transgenic plants; representative staining results are shown here.

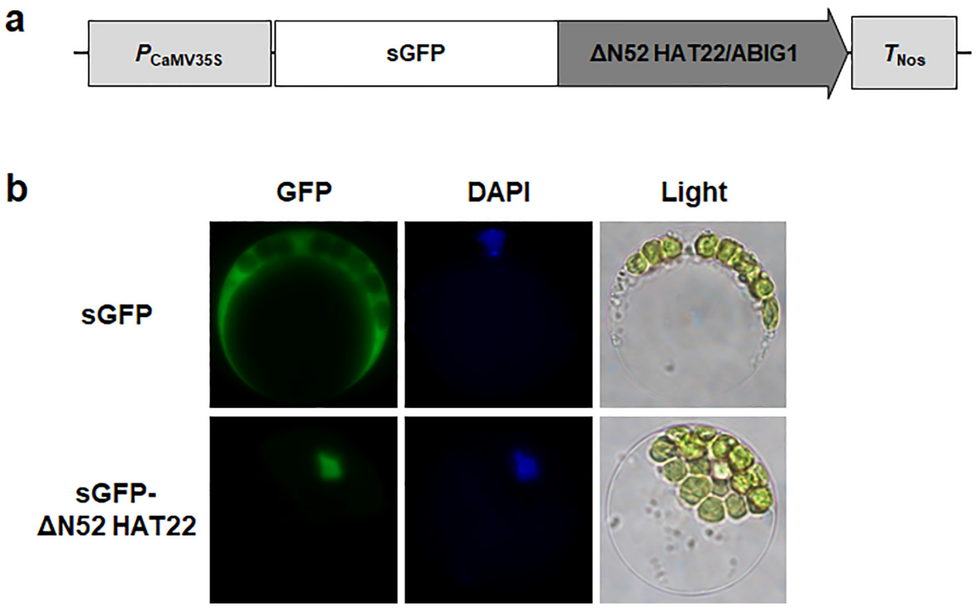

Figure S2. Subcellular localization of ΔN52 HAT22/ABIG1. (**a**) A schematic map of sGFP-fused ΔN52 HAT22/ABIG1 vector. (**b**) Transient expression of sGFP-ΔN52 HAT22/ABIG1 fusion protein in Arabidopsis protoplasts. Left, GFP signal; middle, DAPI staining; right, light microscopy images.

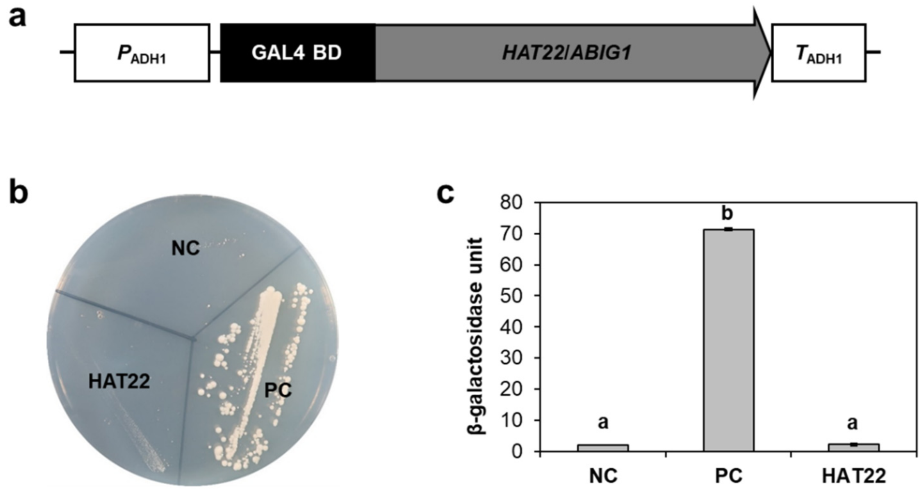

Figure S3. Transactivation activity of HAT22/ABIG1. (**a**) A schematic map of GAL4 BD-fusion HAT22/ABIG1 vector for the analysis of transactivation activity in yeast. (**b**) Yeast growth assay. Yeast transformants were grown on SM-Trp/-Ura. (**c**) Quantitative β-galactosidase ONPG assay. β-Galactosidase activities were used for transactivation activity quantification. Data are shown as means ± S.D. (n = 3). Different letters display significant differences ($P < 0.05$). In (**b**) and (**c**), empty pBD-GAL4 vector and HRE1 were used for the negative and positive controls, respectively.

```
       5′-AGTATT
-110  TGATGTGGGA
-100  GTAGAAATAA
-90   TTAAGAAAGC
-80   GTTATGGTTC
-70   AAATGATAAC
-60   TGCAAATTCT
-50   ATGATTAATT
-40   AACTTATTAT
-30   TATTTATAAG
-20   TTGTTAATGG
-10   TAGTTGCA-3′
```

Figure S4. Identification of HD-Zip II-binding-like *cis*-regulatory element(s) in *HRE2* promoter. Sequence of hypoxia- and salt-responsive region of *HRE2* promoter from -116 to -2 region is presented. The core sequence of HD-Zip II-binding-like *cis*-regulatory element, AATGATA, is marked in red and underline.

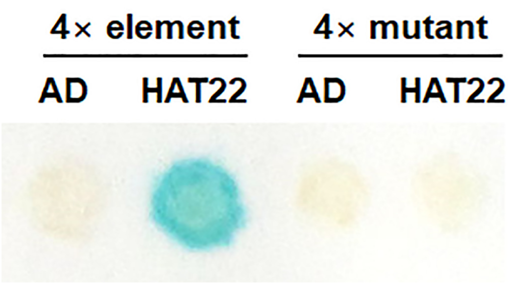

Figure S5. Confirmation of binding of HAT22/ABIG1 to HD-Zip II-binding-like *cis*-regulatory element in yeast. Yeast one-hybrid assay for HAT22/ABIG1 and HD-Zip II-binding-like *cis*-regulatory element or mutant HD-Zip II-binding-like *cis*-regulatory element using β-galactosidase filter assay. 5-bromo-4-chloro-3-indolyl-β-D-galactopyranoside was used as the substrate. The reaction was performed for 6 h.

Table S1. Yeast one-hybrid screening. Results of yeast one-hybrid screens using -116 to -2 region of *HRE2* as bait and hypoxia-treated Arabidopsis cDNA library as prey.

**Total no. of transformants**	**No. of positive colonies**	**No. of isolated genes**
8.8×10^5	25 colonies	13 genes

Table S2. List of genes isolated from yeast one-hybrid screening

Locus ID	Gene	No. of selected yeast colonies	Description
At5g53980	HB52	1	HD-Zip TF family, class I subfamily
At4g37790	HAT22/ABIG1	1	HD-Zip TF family, class II subfamily, subgroup β
At5g47370	HAT2	1	HD-Zip TF family, class II subfamily, subgroup γ
At4g16780	HAT4/HB2/HB-2	1	HD-Zip TF family, class II subfamily, subgroup γ
At3g60390	HAT3	3	HD-Zip TF family, class II subfamily, subgroup δ
At2g44910	HB4/HB-4	1	HD-Zip TF family, class II subfamily, subgroup δ
At2g18350	HB24/ZHD6/ZFHD6	2	ZF-HD
At5g65410	HB25/ZHD1/ZFHD2	1	ZF-HD
At1g75240	HB33/ZHD5	10	ZF-HD
At1g09200	H3.1/HTR2	1	Histone superfamily protein
At4g40040	H3.3/HTR5	1	Histone superfamily protein
At5g10980	H3.3/HTR8	1	Histone superfamily protein
At1g76110	At1g76110	1	HMG box protein with ARID/BRIGHT DNA-binding domain-containing protein

Table S3. List of primers for cloning

Construct	Forward	Reverse
HRE2 promoter -180	5′-GCGAAGCTTTTAGAT AAATTAACTAAAAC-3′	5′-GCGCTGCAGTTTTGT TTTTTGTTTTTTAC-3′
HRE2 promoter -116	5′-GCGAAGCTTAGTATT TGATGTGGGAGTAG-3′	5′-GCGCTGCAGTTTTGT TTTTTGTTTTTTAC-3′
HRE2 promoter -2	5′-GCGAAGCTTAAAGGT TATAGAGCACACAG-3′	5′-GCGCTGCAGTTTTGT TTTTTGTTTTTTAC-3′
HRE2 promoter +52	5′-GCGAAGCTTGAAGAA CTTGGTCGTCAAGC-3′	5′-GCGCTGCAGTTTTGT TTTTTGTTTTTTAC-3′
HRE2 promoter -116 to -2	5′-CGCGTCGACAGTATT TGATGTGGGAGTAG-3′	5′-CGCGAATTCGCAACT ACCATTAACAACTT-3′
HAT22/ABIG1 OX	5′-CGCGTCGACATGGGT CTTGATGATTCATG-3′	5′-ATACCCGGGCTAACA TGCTGCAGAAGGAT-3′
ΔN52 HAT22/ABIG1 OX	5′-CGCGTCGACATGGAGA GCTACAAGATCAAGAC-3′	5′-ATACCCGGGCTAACA TGCTGCAGAAGGAT-3′
GAL4 AD-HAT22/ABIG1	5′-CGCGAATTCATGGGT CTTGATGATTCATG-3′	5′-GCAGGATCCCTAACA TGCTGCAGAAGGAT-3′
GAL4 BD-HAT22/ABIG1	5′-CGCGAATTCATGGGT CTTGATGATTCATG-3′	5′-GTGGTCGACCTAACA TGCTGCAGAAGGAT-3′
sGFP-HAT22/ABIG1	5′-CGCGTCGACATGGGT CTTGATGATTCATG-3′	5′-ATACCCGGGCTAACA TGCTGCAGAAGGAT-3′
sGFP- ΔN52 HAT22/ABIG1	5′-CGCGTCGACATGGAGA GCTACAAGATCAAGAC-3′	5′-ATACCCGGGCTAACA TGCTGCAGAAGGAT-3′

Table S4. List of primers for quantitative RT-PCR

Gene	Forward	Reverse
GAPc	5'-GTGTCCCAACCGTTGATGTC-3'	5'-TCCCTTGAGTTTGCCTTCGG-3'
HAT22/ABIG1	5'-GGTGTGTTCGAGAGTGAGTG-3'	5'-GTTATCTTCGAGAAGAGCAG-3'
RD29A	5'-CCTGAAGTGATCGATGCACC-3'	5'-CAGTGGGTTTGGTGTAATCG-3'
ADH1	5'-TGAATGTGGCTAAACCCAAG-3'	5'-CCTAGAAGCACCAGCGATTC-3'
